# History of nonnative Monk Parakeets in Mexico

**DOI:** 10.1371/journal.pone.0184771

**Published:** 2017-09-19

**Authors:** Elizabeth A. Hobson, Grace Smith-Vidaurre, Alejandro Salinas-Melgoza

**Affiliations:** 1 Santa Fe Institute, Santa Fe, NM, United States of America; 2 Department of Biology, New Mexico State University, Las Cruces, NM, United States of America; 3 Facultad de Biología, Universidad Michoacana de San Nicolás de Hidalgo, Morelia, Michoacan, Mexico; Universite Paris-Sud, FRANCE

## Abstract

Nonnative Monk Parakeets have been reported in increasing numbers across many cities in Mexico, and were formally classified as an invasive species in Mexico in late 2016. However, there has not been a large-scale attempt to determine how international pet trade and national and international governmental regulations have played a part in colonization, and when the species appeared in different areas. We describe the changes in regulations that led the international pet trade market to shift to Mexico, then used international trade data to determine how many parakeets were commercially imported each year and where those individuals originated. We also quantified the recent increases in Monk Parakeet (*Myiopsitta monachus*) sightings in Mexico in both the scientific literature and in citizen science reports. We describe the timeline of increased reports to understand the history of nonnative Monk Parakeets in Mexico. As in other areas where the species has colonized, the main mode of transport is through the international pet trade. Over half a million Monk Parakeets were commercially imported to Mexico during 2000–2015, with the majority of importation (90%) occurring in 2008–2014, and almost all (98%) were imported from Uruguay. The earliest record of a free-flying Monk Parakeet was observed during 1994–1995 in Mexico City, but sightings of the parakeets did not become geographically widespread in either the scientific literature or citizen science databases until 2012–2015. By 2015, parakeets had been reported in 97 cities in Mexico. Mexico City has consistently seen steep increases in reporting since this species was first reported in Mexico. Here we find that both national and international legal regulations and health concerns drove a rise and fall in Monk Parakeet pet trade importations, shortly followed by widespread sightings of feral parakeets across Mexico. Further monitoring of introduced Monk Parakeet populations in Mexico is needed to understand the establishment, growth and spread of introduced populations.

## Introduction

Monk Parakeets (*Myiopsitta monachus*) are small neotropical parrots, notable for their highly social behaviors [1–4], their ability to build nests [1], and their success as an invasive species [5,6]. They are native to temperate southeastern South America [7], and occur mainly in Argentina, Uruguay, Paraguay, and parts of southern Brazil [8]. Monk Parakeets have successfully colonized urban and suburban habitats around the world [7]. Monk Parakeets have been reported in several areas of the United States [5,9–14], many countries in Europe [5,9,15–19], and Israel [20]. They have also been reported outside of their native range in other areas of South America [21]. Monk Parakeets are generally imported into these regions as part of the pet trade [5,22], and then accidentally escape or are intentionally released [23,24]. They tolerate a wide range of environmental conditions [6,25–27], appear to be highly flexible in their nesting and foraging requirements, and are able to tolerate and often even prefer new urban environments [14,28].

Invasive species are of concern especially given the number of negative impacts they may have on native biodiversity [29]. Thus far, studies of the ecological effects of nonnative populations of Monk Parakeets have generally found that the presence of the species has little impact on native bird species [19,30]. While they are highly social, Monk Parakeets generally appear tolerant of other species in mixed foraging flocks, but they will defend their nest sites from other birds [19] and parasite-mediated competition may affect other bird species in some areas [31]. Nonnative Monk Parakeet populations also cause economic impacts, such as damage to structural equipment like electric and communication towers [32–35], as well as crop damage in some areas [36]. Ownership of this species as pets was prohibited in some U.S. states [24,37] due to their economic impact on infrastructure and concerns about feral population establishment and growth. The introduction history of this species around the globe has been different across countries, yet is not well described for some of the more recently colonized areas. Here, we describe the very recent and on-going Monk Parakeet invasion in Mexico.

Monk Parakeets have only recently been reported in Mexico. Based on the timing and location of early sightings, the invasion of the Monk Parakeet in Mexico is most likely the result of an independent invasion process, rather than dispersal from areas where the species was historically reported in the U.S. Following the initial documentation of Monk Parakeets in Mexico, populations have increased drastically, with reports increasing in both the scientific literature and in citizen science databases. However, many of these scientific reports are in Mexican journals and are not easily accessible to non-Spanish speakers, which can impede the international transfer of scientific information [38]. In addition, the large number of observations reported by citizen scientists have not yet been analyzed. Overall, there has not been a systematic country-wide effort to quantify how populations have arrived, appeared, and increased over time. This kind of analysis is needed because Mexico officially classified Monk Parakeets as invasive in the country for the first time in late 2016 [39].

We examine how, why, when, and where Monk Parakeets appeared in Mexico. Because the invasion is fundamentally mediated by the pet trade, we first describe how changes in governmental regulations and both national and international pet trade policies redirected the global pet trade in Monk Parakeets to Mexico. We quantified the changes in numbers of individuals imported into Mexico using data about the international pet trade. We pair data on importation and the timing of regulation changes with observation data from two sources: published reports of Monk Parakeets in the scientific literature and citizen science sighting reports. We use these observations to describe when Monk Parakeets appeared in different regions in Mexico. Our goal is to better understand the history, context, and extent of this recent invasion of Monk Parakeets into Mexico.

## Methods

### International commercial trade data

We obtained data on Mexican imports of Monk Parakeets from Dirección General de Vida Silvestre (DGVS) through the Instituto Nacional de Transparencia, Acceso a la Información y Protección de Datos Personales with request number 0001600402116. DGVS data are reported at the shipment level and include import records from 2000–2015. We also downloaded data on imports and exports of Monk Parakeets from CITES (the Convention on International Trade in Endangered Species of Wild Fauna and Flora) trade statistics derived from the CITES Trade Database, UNEP World Conservation Monitoring Centre, Cambridge, UK (data available at https://trade.cites.org/, accessed 2017-02-28). This dataset contains the total Monk Parakeets reported by two sources: the country that exported the birds, and Mexico, the importing country. Data in this source are pooled by year, and include data from 1975–2015. We use the CITES data for its historical perspective, but rely on the DGVS data to report recent importations and importation at the shipment level within years.

Country-level international trade data are complicated by imperfect reporting which leads to discrepancies in the total number of individuals imported. For example, there may be a difference in the shipment coding, where an exporter did not report the shipment type, but the importer reported the shipment as a commercial shipment (causing reported commercially exported individuals to be lower than reported imports). Even when shipments are coded consistently in reports, the year in which shipments occurred can differ, for example if a shipment is exported in December, but is not recorded as an import until January (see summary of potential discrepancies in [40]). Because of these potential discrepancies, and to provide a comprehensive overview of overall movement of individuals, we report three sources of data on the number of Monk Parakeets imported into Mexico: DGVS reported numbers (reported on importation to Mexico), CITES importer-reported numbers, and CITES exporter-reported numbers.

### Sightings data

We synthesized reports of Monk Parakeet sightings published in the Mexican ornithological literature through the end of 2015 [41–61]. We supplemented these scientific reports with citizen scientist observations (reported through the end of 2015) from several sources: eBird, which launched in 2002 (http://ebird.org/content/ebird/about/), aVerAves, which started in 2004 (but is part of eBird Mexico, and falls under the jurisdiction of the eBird dataset), and iNaturalist, which incorporated in 2011 (https://www.inaturalist.org/pages/about).

We downloaded citizen science reports of Monk Parakeets in Mexico from eBird [62,63]. Because not all sightings were reported to eBird, we also downloaded all Monk Parakeet records reported in Mexico from the GBIF database (GBIF eBird data query with *Myiopsitta monachus* taxon number 2479407, 10.15468/aomfnb, accessed via http://www.gbif.org/dataset/4fa7b334-ce0d-4e88-aaae-2e0c138d049e on 2016-04-11; GBIF iNaturalist data with same query, 10.15468/ab3s5x, accessed via http://www.gbif.org/dataset/50c9509d-22c7-4a22-a47d-8c48425ef4a7 on 2016-04-11).

We then combined all citizen science reports from non-eBird data sources with the directly requested data from eBird. We report data collected between the start of 1999 through the end of 2015 (data from 2016 was not yet fully reported). We excluded 99 records that were duplicated in the GBIF database (i.e. were reported in both aVerAves and iNaturalist datasets). Once duplicates within GBIF were excluded, our database included 1854 records which all had complete date and location information, and were recorded prior to 2016. We determined the city and state for each sighting by reverse geocoding (matching the coordinates of the sighting with spatial data). Some observations in the citizen science database included spatial information, but we checked all records for accuracy. All data were processed, summarized, and visualized in R [64] and mapped using the R package ‘maps’ [65].

## Results

### National and international trade regulations

Several changes in regulations on trade have driven the timing of the Monk Parakeet invasion in Mexico. National and international regulations on trade have undergone recent changes which affected the international pet trade, including trade in Monk Parakeets. Internationally, the EU began to restrict importation of birds from certain countries (starting with southeast Asia) in 2004 due to concerns about the spread of avian influenza [66,67]. This ban was extended to other countries in 2005, when commercial importation of non-poultry birds into the EU was temporarily banned [68,69], and this broader ban on imports was extended again in 2006 [70,71]. Importation of live captive birds to the EU was further restricted in 2007 when the EU announced a permanent ban on imports of wild-caught birds ([72–74] ban in effect 01 July). This ban caused a crash in market demand for Monk Parakeets in the EU.

Following this crash in demand, the international pet market for Monk Parakeets was redirected to Mexico. A change in regulations within Mexico around the same time led to an increase in demand for these imported species. In 2008, the Mexican federal regulations on pet parrot ownership were changed, making it illegal to purchase native Mexican parrot species as pets [75]. With these restrictions, people wanting to purchase legal parrots as pets were restricted to nonnative species, such as the Monk Parakeet. However, in March 2014, the General Directorate of Animal Health, National Service for Agriculture and Food Health, Harmlessness and Quality (SENASICA), notified the World Organization for Animal Health that they had detected avian influenza in a shipment of live Monk Parakeets, imported into Mexico in February 2014 [76]. Despite extensive testing in Uruguay, no evidence for that particular strain of avian influenza was found, and CITES considers the report to be the result of a diagnostic error [76]. However, international importation of Monk Parakeets into Mexico ceased following the report, and no individuals were imported in 2015 (see below).

### International commercial trade

The CITES trade dataset includes data from 1975–2015, so we used the CITES dataset for a historical perspective on overall Monk Parakeet trade with Mexico. According to CITES, 531670 live Monk Parakeets were reported exported to Mexico, while 591313 live Monk Parakeets were reported imported into Mexico for commercial purposes between 1975 and the end of 2015. There is a discrepancy of 59643 individuals between the numbers of individuals reported by countries exporting to Mexico and the number of individuals reported imported into Mexico in the CITES dataset.

The earliest reported live commercial trade in Monk Parakeets to Mexico in the CITES dataset occurred in 1981, when 235 individuals were reported exported to Mexico. However, this report was recorded on the CITES exporter-report only (CITES importer-reports report 0 individuals imported in 1981). Mexico did not report any live commercial imports of Monk Parakeets until 1994 (when 10 individuals were reported imported to CITES). Between 1981 and 2000, importation numbers were very low, with between 1167–1494 live individuals commercially imported (numbers vary based on source of report, see [40]). Compared to overall CITES reported trade 1975–2015, trade during 1981–2000 accounted for just 0.2% of total imported individuals.

Between 1975 and 2000, individuals imported to Mexico originated from just 4 countries (CITES importer-reported data). The vast majority of historically imported birds (1975–2015) originated from Uruguay (97%, 574060 individuals), while 3% came from Argentina, less than 1% were from Paraguay, and just 15 individuals were reported imported from Belgium (an area outside the native range).

**Fig 1:**
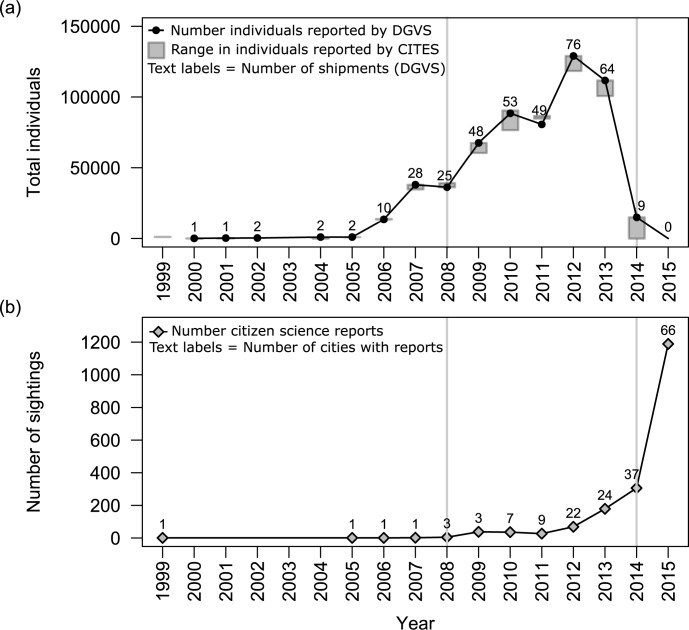
Importation and feral sightings of Monk Parakeets in Mexico (1999–2015). Panels show (a) import data on the number of individuals imported: CITES data is plotted as the range of importer-reported numbers and exporter-reported numbers, import data from DGVS is plotted as points, number of shipments reported by DGVS are included as text labels over points; and (b) number of citizen science reports of feral Monk Parakeets, with the number of unique cities with citizen science reports as text labels over points. Vertical grey lines show the years in which Mexican pet parrot regulations changed (2008) and when international importation of Monk Parakeets ceased (2014).

We then compared the CITES dataset with DGVS data from 2000–2015. Like the CITES importer-reported data, DGVS reports more individuals (52870) imported than are reported in CITES exporter-reported data (2000–2015). In contrast, importer-reported numbers obtained from DGVS were much more consistent with CITES importer-reported data, although there were more minor discrepancies in overall imports (see Fig 1A). There were 7100 more individuals reported imported live for commercial purposes by CITES importer-reports than in DGVS data. Import numbers from DGVS match CITES importer-reported numbers for 11 of the 15 years reported by DGVS (73%). DGVS reports are slightly higher than CITES importer-reported numbers in 2002 and 2004 (by 360 and 500 individuals respectively) while CITES importer-reported numbers were higher than DGVS reports in 2010 (by 1960 individuals). The largest discrepancy was in 2011 when CITES importer-reported amounts were larger than DGVS reports by 6000 individuals.

Because the DGVS dataset contained additional information on individual shipments which the CITES dataset did not include, we used the DGVS dataset to quantify the total number of individuals imported and the number of shipments during 2000–2015. Between 2000 and 2015, 583046 live Monk Parakeets (in 370 shipments) were commercially imported into Mexico according to DGVS data. The vast majority of these imports originated from Uruguay (97%, 566100 individuals, 349 shipments). Argentina exported an additional 3% (16186 individuals, 17 shipments) and with the remaining <1% (760 individuals, 4 shipments) exported by Paraguay. Between 2008 and 2014, 98% of imported individuals originated from Uruguay, 2% from Argentina, and no individuals were imported from Paraguay.

Of the total individuals reported imported in CITES data between 1975 and 2015, 90–91% of these imports (depending on reporting source) took place between 2008 and 2014. Due to concerns about an avian influenza contaminated shipment, importation into Mexico ceased during 2014 [76]. No live Monk Parakeets were commercially imported into Mexico in 2015.

### Observational reports

**Fig 2:**
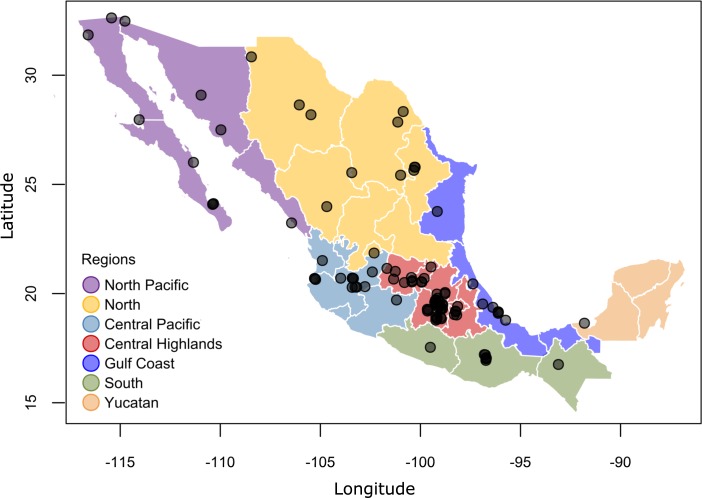
Mexican cities with reported Monk Parakeet sightings, by region. Points indicate cities in which at least one reported sighting was entered into online citizen science databases or in a published scientific report, during 1999–2015. Mexico state shapefiles were downloaded from the GADM database of Global Administrative Area (version 2.8, available at shttp://www.gadm.org/download, Mexico, level 1) and mapped using the R package ‘maps’ [65].

We found 21 published references to Monk Parakeet sightings in Mexico published between 1999 and 2015. In citizen scientist databases, we used 1854 sightings of Monk Parakeets reported in Mexico from 1999 through the end of 2015 (Fig 1B). We divided Mexican states into 7 geographic regions to consider region-specific invasion patterns (Fig 2). Combining the scientific reports and the citizen science database, Monk Parakeets have been reported in a total of 97 cities, and have been observed in all 7 geographic regions (Fig 2).

**Fig 3:**
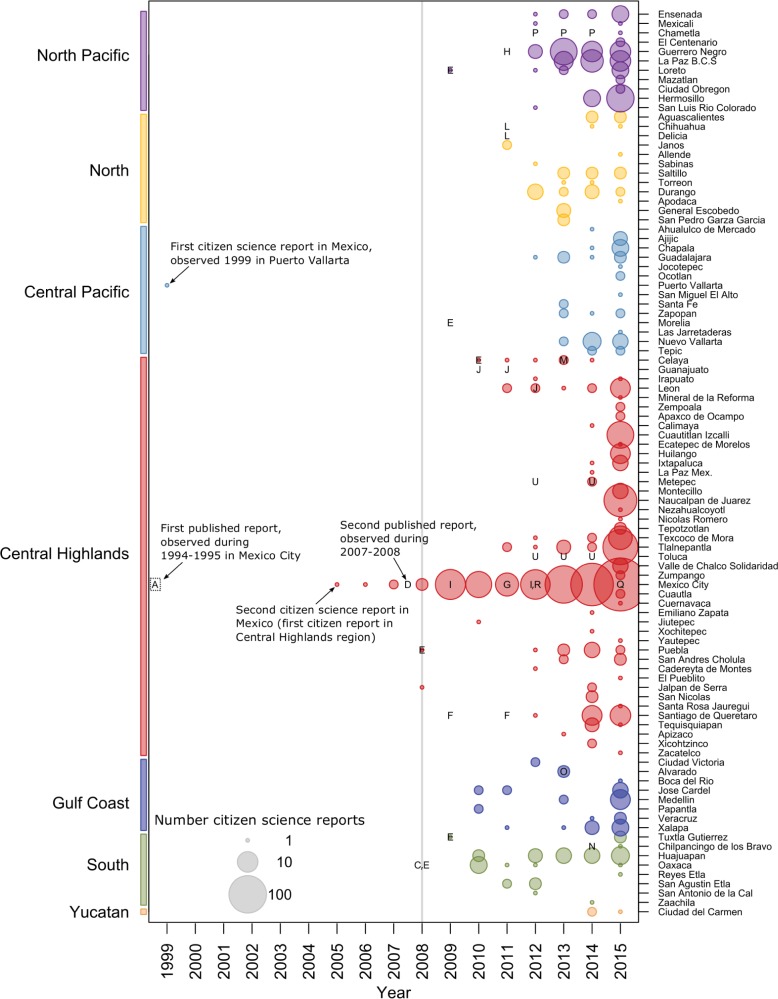
Monk parakeet reports in time and space. Text labels and colored bubbles indicate reports of Monk Parakeets in cities during particular years; text labels indicate a report in the scientific literature, and is linked to the citation (see below), while bubbles indicate citizen science reporting (colored by region and log-scaled by number of reports made in a particular city in a particular year). For both citation text labels and citizen science bubbles, the location at which they are plotted indicates the city and year in which observations occurred. Thus, Monk Parakeets may have been reported in just the scientific literature in a city during a year (only text label), reported in just the citizen science database (only colored bubble), reported in both the scientific literature and by citizen scientists (text label and bubble), or not reported at all (blank). Each text label corresponds to a particular citation in the scientific literature, as follows: *A* [41], *B* [42] (not plotted, lack of information about observation), *C* [43], *D* [44], *E* [45], *F* [46], *G* [47], *H* [48], *I* [49], *J* [50], *K* [51] (not plotted, year of sightings is unclear), *L* [52], *M* [53], *N* [54], *O* [55], *P* [56], *Q* [57], *R* [58], *S* [59] (not plotted, year of observation not specified), *T* [60] (not plotted, years of study listed as 2009–2015, but years of parakeet observations not specified), *U* [61]. The grey vertical line indicates the year the ban on pet trade of native parrots was passed.

In the scientific literature, the earliest published report of Monk Parakeets in Mexico occurred in Mexico City, where the species was sighted during a biodiversity study sometime during 1994 and 1995 (exact date not reported, [77]). Citizen science reports from early years are uncommon, as most of the reporting services started relatively recently (e.g. eBird started in 2002). The first citizen science report of a Monk Parakeet in Mexico occurred in 1999, when a single individual was sighted in Puerto Vallarta and reported on eBird (observation back-dated 1999-Nov-29). These two sightings were almost certainly independent, as Mexico City and Puerto Vallarta are over 800km apart. Monk Parakeets were not reported again in Puerto Vallarta in the citizen science data (1999–2015), and were not reported in Mexico City again in either published scientific reports or citizen science databases until 2005 (see Fig 3). These two early sightings are most likely isolated reports, rather than indicative of sustained occupation.

During 2005–2007, both citizen scientist reports and published accounts in the scientific literature report Monk Parakeets exclusively in Mexico City. Citizen scientists reported 4 observations in Parque Ecológico de Xochimilco. All citizen observers report either a single individual, or did not specify group size, until September 2007, when a flock of 6 birds was reported in this area. At some point between 2007 and 2008, at least one Monk Parakeet was observed in Mexico City and reported in the scientific literature (see Fig 3, text label “D”), but the date of observation was not specified [44].

Citizen scientists reported Monk Parakeets in just 3 cities in 2009 (Fig 1B). After international and Mexican trade regulations were changed, and Monk Parakeets were more commonly imported (see above), both the total number of Monk Parakeet reports (Fig 1B) and the geographic range of observations (Fig 2) increased in both the scientific literature and citizen scientist databases. Between 2011 and 2015, the number of cities with Monk Parakeet reports increased dramatically, from 9 cities in 4 of 7 geographic regions, to 66 cities across all 7 geographic regions (Fig 3). By the end of 2015, Monk Parakeets had been sighted at least once in 97 cities in Mexico, as reported in the scientific literature and citizen science records. During 2015, Monk Parakeets were sighted in 68% of these cities.

### Geography of invasion

The Central Highlands region, which includes Mexico City, has the longest history of Monk Parakeet sightings, the highest number of cities with sightings compared with the other geographic regions (Table 1), and the most consistent yearly reporting of parakeet sightings (Fig 3). The presence of Monk Parakeets in other regions is more recent and less widespread: the earliest citizen science reports of Monks in the Yucatan region for example date to 2014, and include just one city in that region (Table 1). Citizen science reports from cities in these regions are also less consistent year to year. Whether this is indicative of a non-persistent population and subsequent appearance of new individuals, or whether this is a reporting issue, is difficult to definitively determine with the current data.

**Table 1:** Citizen science reports summarized by geographic region.

Region	Number reports	Number cities	Earliest	Latest	Years with reports
North Pacific	140	11	2009	2015	5
North	44	11	2011	2015	5
Central Pacific	51	13	1999	2015	5
Central Highlands	1526	41	2005	2015	11
Gulf Coast	44	8	2010	2015	6
South	46	8	2009	2015	7
Yucatan	3	1	2014	2015	2

## Discussion

We reconstructed the history of the Monk Parakeet invasion in Mexico using a combination of international trade data, the timing of national and international regulation changes, and observational reports from the scientific literature and citizen science databases. These multiple sources allowed us to describe the history of nonnative Monk Parakeets in Mexico, and sets baseline occupancy observations for the country.

We used international trade data to determine how many parakeets were imported each year into Mexico, and where these individuals originated. As in other areas where the species has colonized, the predominant mode of transport was through the international pet trade. Over half a million Monk Parakeets were commercially imported to Mexico during 2000–2015, with the majority of importation occurring 2008–2014, and most of these were wild-caught birds from Uruguay.

This increase in historical importation levels roughly coincided with two events. First, the increase in importation to Mexico began after the importation of wild-caught birds in Europe was restricted in 2005 [69,70], and permanently banned in 2007 [74], due to concerns about the spread of avian influenza. Given the timing of the increase in Monk Parakeet importations to Mexico, this suggests that the EU regulation change may have caused the international pet trade in Monk Parakeets to be redirected from European to Mexican markets. Second, a 2008 change in Mexican governmental regulations restricted the types of parrots allowed in the pet trade, which redirected demand in the legal pet trade within Mexico for nonnative parrot species, and increased demand for nonnative Monk Parakeets. The intent of the ban on native parrots in the pet trade was to protect native Mexican species from overharvesting [75], especially in cases where parrots are illegally captured in the wild and then sold in the pet trade. As such, it serves an important protective function for the many threatened and endangered parrots in Mexico. Recent reports also suggest that the ban is working. The Procuraduria Federal de Proteccion al Ambiente (PROFEPA) reported in March 2017 that the ban on native parrots led to a 24% decrease in illegal trafficking activity in parrot species following the regulation change [78].

Like Europe, Mexico responded to concerns about the spread of avian influenza via international trade. Commercial importation of Monk Parakeets stopped in 2014 due to concerns about a potential avian influenza contamination in a shipment. Europe responded earlier to avian influenza concerns (partial restrictions beginning in 2004), while the import restrictions in Mexico are more recent (2015). Also, unlike Europe, Mexico did not implement a blanket ban on wild bird imports; the cessation in importation was a response to concerns about the Monk Parakeet specifically. However, as we detailed above, CITES considers this report of avian influenza in the Monk Parakeet shipment to be a diagnostic error [76], rather than indicative of an actual outbreak.

The changes in European international regulations and Mexican national regulations, which increased importation, and Mexican health concerns, which stopped importation, created a short window of time during which large numbers of parakeets were imported and entered the pet trade in Mexico. This importation pulse resulted in an increased propagule size, where a large number of individuals had the potential to colonize Mexican cities due to intentional releases or to accidental escapes from captivity. Propagule pressure may be a factor influencing the success of an invading species [79,80]. The largest number of feral Monk Parakeet sighting reports occurred in 2015, one year after the ban on international importation of Monk Parakeets. Whether this lag is indicative of a delay in birds escaping captivity is unknown, as information does not exist regarding the average time Monk Parakeets are kept in captivity by pet owners, nor the propensity for Monk Parakeets to escape or be intentionally released in Mexico. In other areas, escape from captivity has increasingly been seen as a prominent source of species introductions, especially for vertebrates [81,82]. Pet parrot escapes in particular were found to be more frequent than previously acknowledged in a study conducted in Australia [81].

We found that the increase in reported sightings of Monk Parakeets roughly coincided with the changes in national and international regulations, and the associated increase in international commercial importation. The earliest record of a free-flying Monk Parakeet was observed during 1995–1996 in Mexico City. After this early sighting, two isolated sightings of parakeets were reported: in Puerto Vallarta in 1999 and a second report in Mexico City in 2005. However, it was not until 2011–2015 that reports of Monk Parakeets became geographically widespread in Mexico. By the end of 2015, Monk Parakeets had been observed in 97 cities across all regions of Mexico. These increasing reports of sightings throughout Mexico could indicate a constant nationwide propagule pressure, which may have been stronger in Mexico City due to the larger overall human population size.

While these reports can provide information on locations where Monk Parakeets may have colonized, this increase in reporting does not necessarily correlate with increased feral population sizes. This is because citizen science reporting may have increased in frequency or popularity, or more people could be reporting the same individual parakeets in a particular neighborhood. More information is needed on population sizes in cities where Monk Parakeets have been observed in order to more accurately estimate current feral populations. We also cannot conclusively determine whether populations in particular cities are well established. In invasive species biology, a species is generally considered “established” when individuals are consistently present and the local population is surviving and reproducing (e.g. [83]). The inconsistency that we found in year to year observations at the city level could indicate that Monk Parakeets did not have established populations in at least some cities in Mexico. Cities where the parakeets are consistently reported across consecutive years are more likely to host established populations, but more information on reproductive success is needed. More information is also needed on the habitat preferences of Monk Parakeets within these regions. Invasive Monk Parakeet populations worldwide exploit and often seem to prefer urban habitats [14,28], and few invasive populations have expanded into more rural habitats. Because the Mexican invasion is so recent, it is difficult to predict whether they will expand into more rural areas in Mexico, but this seems unlikely given the invasive habitat preferences in other areas.

With our analysis, we also cannot determine whether populations in different cities were independently founded from within-city escaped or released individuals (e.g. founded directly from ex-pets which were wild-caught in the native range and transported to a particular city where they escaped), or whether nearby cities were colonized by feral individuals from neighboring populations (e.g. founded by secondary movement of feral birds from their area of introduction, or by subsequent generations of introduced populations). There is currently no direct evidence of long-range dispersal among cities in the non-native range, but genetic analyses suggest it may occur [84]. In contrast, in the native range, limited data on juvenile dispersal suggests that young individuals generally do not disperse far from their natal areas [85].

Our work describes the history of Monk Parakeet feral populations in Mexico, and sets baseline occupancy observations for the country. Whether feral Monk Parakeet populations in Mexico are established and capable of sustained persistence and growth is an open question, and the subject of some of our team’s future work. Following the 2014 ban on commercial imports to Mexico, feral Monk Parakeet populations may gradually decline due to decreased propagule pressure, unless populations have become established and are capable of reproducing. Monk Parakeet flocks in areas such as Mexico City, which have been observed and reported for several consecutive years, are more likely to represent established populations. In other cities with fewer reports, it is difficult to determine whether populations are firmly established.

Continued monitoring of the Monk Parakeet in Mexico is necessary to better understand the invasion dynamics. Other than the current ban on international importation, the species has not yet been federally managed or regulated by the Mexican government. Monk Parakeets were officially listed as an invasive species in Mexico in late 2016, and government regulations require the implementation of management actions. Because the species was so recently listed, no management action has been undertaken yet. If management actions are undertaken, this study could serve as a baseline comparison to evaluate the success of the implementation of these actions. However, prior to forming any management plan, more study is needed to better determine the impacts of Monk Parakeets on native species, urban infrastructure, and local economies.
